# 

**Published:** 1911-02-04

**Authors:** 


Just Issued. Ninth Edition, Rewritten and Enlarged, 9s. net.

**PHARMACY, MATERIA MEDICA & THERAPEUTICS.**

Being a Commentary on the B.P. and its Drugs, with their Preparations, and on all the new Non-official Remedies in use, with Chapters on the different processes used in Dispensing, Prescription-writing, Latin Glossary, &c. By Sir W. WHITLA, M.D., LL.D., Professor, Materia Medica, Queen's University, Belfast, and Senior Physician, Royal Victoria Hospital.

By the same Author. New Work. 1,900 pages. Crown 8vo. 25s. net.

**PRACTICE OF MEDICINE.**

Containing in two Handy Volumes a complete and independent Article on the Etiology, Symptoms, and Diagnosis of every Medical Disease; arranged in Dictionary Form for Practitioners and Students.

London: BAILLIÈRE, TINDALL & COX, 8 Henrietta Street, W.C.

**Suppurative Pericarditis.**

The symptoms are often exceedingly vague, and in childhood it is most difficult to lay the correct stress upon such as are present, for two reasons. One is the usual absence of pericardial friction, the other the almost invariably co-existing pulmonary disease, which draws the attention away from the heart.—*Dr. F. J. Poynton.*

**Infantile Scurvy.**

A mixture or alternation of potato pulp, fresh milk, and raw meat juice will generally be found useful and acceptable to the child. A fine potato gruel, made by beating up with milk the floury parts of steamed potatoes. A teaspoonful of this creamy gruel may be added to each bottle of food. Well-boiled carrots may be treated in the same way, or freshly made beef tea or chicken broth may be used instead of milk. The child should also be given a teaspoonful of grape or orange juice occasionally.—*Dr. Burney Yeo.*

### Obituary.

The death is announced at Pará, Brazil, of Mr. Walter Myers, M.A., M.B. Cantab., "from yellow fever contracted whilst working for the Liverpool School of Tropical Medicine. In his twenty-ninth year."

The death is announced of Mr. Frederick Melland, M.R.C.S., L.S.A., in his ninety-third year, at Manchester. His membership of the Royal College he gained in 1841, and his L.S.A. the year previously.

News has come from Malta of the death of Fleet-Surgeon James Macdonald Rogers, R.N., F.R.C.S., of H.M.S. *Orontes*, aged fifty-four. Mr. Macdonald took his L.S.A. in 1880, studied at the Middlesex Hospital, became M.R.C.S. in 1881, and F.R.C.S. three years later.

Mr. Justin Theodore Vulliamy, of Rugby, has died at the age of forty-two. Mr. Vulliamy, who, we believe we are correct in stating, was a connection by marriage of George Meredith, studied at St. Thomas's Hospital, and took his diploma in 1897. He was afterwards medical officer to the Rugby Hospital, Surgeon to the Provident Dispensary there. His other appointments included that of resident house physician to the Brompton Hospital for Consumption, and also to the French Hospital. He had formerly been assistant house surgeon to the Lincoln Country Hospital.

The death is announced at Dawlish of Dr. Matthew Coates, retired Deputy Inspector-General of his Majesty's Hospitals and Fleet. Dr. Coates was educated at Bristol and Paris and Durham, took his M.R.C.S. in 1859, and in 1869 became a Fellow of the Royal College, and obtained the M.D. degree at Durham University in 1883. He was an Honorary Associate of the Order of St. John of Jerusalem. Dr. Coates began his naval career as a surgeon in 1859, was promoted to the rank of staff-surgeon in 1871, and as a fleet surgeon in 1882, when he retired with the honorary rank of Deputy-Inspector-General of Hospitals and Fleets. His appointments included that of assistant surgeon to the Royal Naval Hospital, Haslar, from 1863-65, and to H.M. Dockyard, Devonport, from 1867-71, and surgeon in charge of the Royal Naval Hospital, Esquimalt, Vancouver Island, from 1875-78. On his retirement in 1882 Dr. Coates practised at Streatham, and was appointed medical officer to the local post-office. He was one of the organisers of the St. John Ambulance Association, and was honorary surgeon and lecturer to the brigade. He also lectured on ambulance work to the late London School Board, and was honorary surgeon to the Redhill Philanthropic Association.

The death took place last week at his house at Whitby of Mr. Henry Power in his eighty-first year, late consulting ophthalmic surgeon to St. Bartholomew's Hospital and the Westminster Ophthalmic Hospital. Mr. Power, who had retired from practice, was the son of the late Lieutenant-Colonel John Francis Power, King's German Legion, and was educated at Cheltenham College and St. Bartholomew's Hospital, became M.R.C.S. in 1831 and a Fellow of the College in 1854. The next year he gained the M.B. of London University, being also University medical scholar in surgery and comparative anatomy. He at one time was President of the Ophthalmic Society and Vice-President of the Royal College of Surgeons, to which he had been also Professor of Surgery and Arris and Gale Lecturer on Anatomy and Physiology. He was the author of "Elements of Human Physiology," "Illustrations of the Principal Diseases of the Eye," and was editor (with Dr. Sedgwick) of "Mayne's Expository Lexicon."

If the suggestion and cheque for £500 of an anonymous gentleman be accepted Chiswick will have a memorial to King Edward of its own. The cheque is conditioned by the adoption of a cottage hospital as the form of the memorial. It will be remembered that the County of Middlesex has received an appeal from the Lord Lieutenant, the Duke of Bedford, for a similar purpose, though a hospital is not the form which the county is asked to adopt.

Dr. Frederick Langmead has been appointed as Assistant Physician to the Royal Free Hospital, Gray's Inn Road, W.C., to fill the vacancy caused by the resignation of Dr. Farquhar Buzzard.

### Gifts and Bequests.

Prince Henry of Battenburg has sent a gift of game to the Belgrave Hospital for Children.

Lord Sandhurst, Treasurer of St. Bartholomew's Hospital, acknowledges £50 from "A Governor."

The Royal Hospital for Incurables, Putney Heath, has received fifteen guineas from the Carpenters' Company and 50 guineas from the Clothworkers' Company.

Prince Alexander of Teck has received from the trustees of the late Mlle. Louise Bellevue £500, and from Miss O. C. Snell £315, towards the Prince Francis of Teck Memorial Fund.

Mr. C. S. Robinson has given to the Leicester Infirmary a hundred collecting boxes of the same pattern as those now used in the savings banks.

Mr. William Hathornthwaite, of Salop, has bequeathed £100 each to the National Anti-Vivisection Society and to the National Anti-Vivisection Hospital, Battersea.

The Merchant Taylors' Company has voted £1,000, payable in two annual instalments of £500 each, towards the special fund for the reduction of the capital debt of St. Bartholomew's Hospital.

Miss Julia Maria Sterling, of Falmouth, a well-known philanthropist in the district, has left £4,000 to Mrs. Leila Parnell, or, if dead, to Miss Alice Gregory, for hospital work; and £50 to the Budock Nursing Fund.

Miss Hampson, of Radcliffe, has left £1,000 each, free of legacy duty, to the Manchester Royal Infirmary, the Bury Infirmary, the Southport Infirmary, the Pendlebury Hospital for Children, and to the trustees of the Radcliffe and Whitefield Consumption Fund.

The King has sent gifts of game to St. Thomas's Hospital, Guy's Hospital, University and King's College Hospitals, the West London Hospital, Hammersmith Road, the Charing Cross, Westminster, and Cancer Hospitals, the Hospital for Women, Soho Square, and St. Mary's Hospital, Paddington.

Lady Hambleden has just sent £100, her fifteenth donation, to King's College Hospital Removal Fund. Other recent donations include £1,000 from Mr. Mortimer Singer to name a bed in the new hospital, and a further £1,000 from Mr. F. C. Yates. The latter have been sent in response to the special appeal that is being made in connection with the forthcoming dinner on behalf of the fund.

Towards the £150,000 proposed to be raised in Leeds for the extension and improved equipment of its General Infirmary in that city four sums of £5,000 each have been subscribed or promised (in addition to Lord Airedale's contribution) by the Hon. Edward Wood, M.P., Mr. Joseph Watson (with an additional £1,000 when £100,000 is raised), Mr. Walter Cliff, and Mr. Stephen Cliff; Mr. J. W. Wilson, Mr. Samuel Wilson, and Mr. Gladstone Wilson have together given £5,000.

Mr. Lewis Randle Starkey, of Norwood Park, Southwell, Notts, Conservative M.P. for the Southern Division of the West Riding of Yorkshire, 1874-80, lately a director of the Midland Railway Company, and of Messrs. Starkey Brothers, Ltd., woollen manufacturers, of Huddersfield, who left estate valued at £200,310 gross, has bequeathed £250 to the Newark General Hospital and £100 to the Huddersfield Infirmary, these bequests being payable if he should not have made an equivalent donation between the date of his will (August 31, 1909) and the date of his death.

Mrs. Martha Anne Walton Wise, of Tonbridge, has left £50 to the Tonbridge Cottage Hospital.

Miss Elizabeth Clotilde Hoare, of Fareham, Hants, has left £500 to the Royal Southampton Hospital.

The late Mr. George Reid has left £1,000 to the West Fife Hospital towards the building of a convalescent home in connection with the hospital.

The Council of the Amalgamated Hampstead General and North-West London Hospitals have received an anonymous £1,000 for the rebuilding of the out-patient department (North-West London Hospital), Kentish Town.

Major-General Blair Thomas Reid, of Branksome Park, Bournemouth, has left £300 to the Victoria Hospital, Bournemouth.

Mary Lady Tollemache, of Helmington, Stowmarket, has presented a Vernon Harcourt chloroform inhaler to the East Suffolk and Ipswich Hospital through Mr. W. W. Sinclair, the hon. ophthalmic surgeon to the hospital.

Mrs. Sarah Jenkins, of Edgbaston, Birmingham, has left £500 each to the Royal Orthopædic and Spinal Hospital, Birmingham, and the Birmingham and Midland Free Hospital for Sick Children; £350 to the Birmingham and Midland Skin and Urinary Hospital; £250 each to the Birmingham General Hospital, the Queen's Hospital, Birmingham, the Birmingham and Midland Hospital for Women, the Birmingham Lying-in Charity and Maternity Hospital, the Birmingham and Midland Counties Sanatorium, Blackwell, Worcestershire, and the Birmingham and Midland Eye Hospital; £200 each to the Birmingham District Nursing Society, the Moseley Hill Convalescent Home for Children, near Birmingham, and the Dental Hospital, Birmingham.

Miss Juliet Susannah Leo, of Upper Norwood, S.E., formerly of Manchester, has bequeathed £1C0 each to the Norwood Cottage Hospital, the Streatham Home for Incurables.

Captain Matthew Henry Foster, of Seacombe, Cheshire, retired master mariner, an associate of the Institute of Naval Architects, has left £1,000 to the Victoria Central Hospital, Liscard, to endow a "Matthew Henry Foster" bed in that hospital.

Dr. Joseph Frank Payne, M.D., F.R.C.P., of Lyonsdown House, New Barnet, Herts, Hon. Fellow of Magdalen College, Oxford, Emeritus Librarian to the Royal College of Physicians, who left estate valued at £15,285 gross, has left £30 to the Samaritan Fund of St. Thomas's Hospital, and to the Royal College of Physicians his manuscripts of Guibertus Anglicus, Nicolaus Prepositus, Nicolaus Falcuttus, "De Simplici Medicina," attributed to Roger Bacon, "A Choyce of Sundrie Medicine," by Nathaniel Butler, and "Receptes Medicinales" in English, French and Latin; and his books "Bücherkunde für Aeltere Medizin," by Choulant, and "The New Cure of Fevers," by Andrew Bacon.

---

Captain Alfred Hutton, of Jermyn Street, and formerly of Beverley, Yorks, a founder of the Throat and Ear Hospital, and one of the finest swordsmen in Europe, who died on December 18 last, aged seventy-one, has left estate valued at £15,556 gross. Captain Hutton was a man of diverse interests, of a type getting rarer now about St. James, but which in the sixties had a curious survival from the glories of an earlier age. He was President of the Amateur Fencing Association, author of numerous works on fencing and weapons, and the pioneer of bayonet fencing in the British Army.

### Jottings.

Mr. Hugh Gavin, of Arbroath, has been appointed architect of the proposed reconstruction of Arbroath Infirmary, which has been decided on as the result of Dr. D. J. Mackintosh's report.

It has been decided at a town's meeting held at Romford, Essex, to build an addition to the local hospital as a memorial to King Edward. The hospital was originally erected in commemoration of the Jubilee of Queen Victoria. On the completion of the new building the institution will be called the Victoria and Edward Hospital.

The council of the Chelsea Hospital for Women states, for the information of any who may receive unauthorised appeals, that they have discontinued the use of collecting-boxes outside the hospital precincts.

The Mayor of Bexhill presided at a representative public meeting to consider the question of establishing a hospital as a fitting memorial to King Edward VII. After considerable discussion an influential committee was appointed to report at a future public meeting.

A Local Government Board inquiry has been held into the application of the Earlestown Newton District Council for sanction to borrow £5,400 for the provision of an isolation hospital.

The sanction of the Local Government Board for a loan of £2,267 for isolation hospital buildings at Newark has been asked by the Urban District Council.

The will of the late Mr. W. H. Christie provides for a substantial legacy to the East Sussex Hospital. The question of removing the hospital to a more advantageous site above Hastings has recently been discussed as a fitting memorial to the late King.

Foundation-stones have been laid for a new hospital at Gainsborough, the funds for which have been provided under the will of the late Mr. Coupland, who left about £50,000 for the purpose. A scheme is being prepared for an annexe to the hospital to contain an installation of the Röntgen Ray apparatus.

The Maharaja of Mysore has laid the foundation-stone of an ophthalmic hospital in the city of Bangalore by way of commemorating the Viceroyalty of the Earl of Minto.

Lord Spencer (the Lord-Lieutenant of Northamptonshire) has addressed a letter to the mayors of boroughs, the chairmen of urban district councils, the chairmen of parish councils, and the chairmen of parish meetings in the county appealing for contributions to a fund for a county memorial to King Edward. He suggests that the fund should be devoted to some hospital.

The Maharajah of Patiala has contributed a lac of rupees, in addition to other valuable gifts, to the King Edward Sanatorium for Consumptives at Dharampore. The Maharajah is also making arrangements for the erection of an up-to-date dispensary at the same place for the treatment of both out- and in-patients.

A Red Cross Hospital is to be built at Bangkok, says the correspondent of the *Times*, as a memorial to the late King Chulalongkorn, whose children have subscribed over 100,000 ticals (a tical=1s. 6½d.) to the Red Cross Society for the erection of the hospital. The main building will be open to all nationalities.

A concert to aid the Middlesex Hospital funds, as a memorial to Prince Francis of Teck, was given by desire of Prince Alexander and with the approval of their Majesties in the Queen's Hall on January 24, when Verdi's "Requiem" was sung by the Brighton Choir. The soprano part was taken by Miss Alys Bateman, and other performers were Miss Hannah Jones, Mr. Ben Davies, and Mr. Edmund Burke.

The West Fife Hospital has created a record in its history by having during 1910 a daily average number of occupied beds amounting to 56.5.

The foundation-stone of the new Hospital at Gainsborough, near Hull, was laid recently by Mrs. Cross, whose first husband, Mr. Coupland, left about £50,000 for the institution.

Mr. Councillor Booth is reported to have stated at the annual meeting of the Rochdale Infirmary Workmen's Committee that the place was frightfully crowded, and that the infirmary's operating theatre was a disgrace to the town.

Two cots, the gift of the late Mr. Alderman C. Cameron Walker, J.P., have been dedicated at the York County Hospital. The late alderman always took a kindly interest in poor children, particularly in those who were inmates of the workhouse.

There are now 232 in-patients at the Royal Hospital for Incurables, Putney Heath, which is the largest number since the institution was founded in 1854. In addition to this, there are 700 pensioners, who are all incurable invalids, and each of whom is in receipt of the pension of £20 a year.

The Government of Madras, after examining in detail the proposals and estimates of a committee appointed in August 1910 to consider the question of establishing one or more sanatoria for consumptives in the Madras Presidency, has come to the conclusion that they are too ambitious for adoption in the present circumstances, having regard to the funds available from public subscriptions to the Edward VII. Memorial and otherwise. It considers, however, that the following are measures which call for adoption : (*a*) The establishment of a sanatorium in the southern part of the Presidency, preferably at or near Coimbatore, where consumption is most prevalent; (*b*) the erection of temporary phthisis wards in a few specially selected district headquarters hospitals; (*c*) grants of assistance from public funds to the sanatorium which the Mission of South India proposes to establish at Madanpalle.

The new wing at the Weston-super-Mare General Hospital, which was "opened" just before Christmas, is now in working order. The gift of Mr. Hooker, a descendant of the author of the "Ecclesiastical Polity," this wing fulfils the local need for an isolation block, and the architects, Mr. Hans Price and Mr. William Jane, have been congratulated upon it. The block is appreciated locally all the more because it replaces a heap of buildings which had not even the charm of antiquity to plead as a justification for other defects that were indeed obvious.

---

The following appointments have been approved at the General Infirmary, Leeds : Mr. J. Perrin Brown, M.B., honorary surgeon to Mr. Moynihan; Mr. C. G. K. Sharpe, M.B., honorary surgeon to Mr. A. L. Whitehead; Mr. A. E. Taylor, M.B., honorary surgeon to Mr. Thompson; Mr. G. V. Stockdale, M.B., honorary physician to Dr. Griffith; Dr. H. J. McVean, assistant honorary anæsthetist.

### TO HOSPITAL SECRETARIES AND OTHERS.

We invite contributions from any of our readers, and shall be glad to pay, according to length, for items of news for the institutional section (including appointments, resignations, gifts, etc.) which we may publish. All payments for manuscripts used are made as early as possible after the beginning of each quarter.

### BUILDING WORKS CONTEMPLATED AND IN PROGRESS.

The rebuilding of the out-patient department of the North-West London Hospital, Kentish Town, is to be undertaken.

The King Edward Memorial Fund for the removal of Hastings Hospital has reached £7,000. The Mayor has given fifty guineas, and will double this if the new building be begun during the year.

Seventy architects are at work already on competitive plans for the reconstruction of Bradford Royal Infirmary. Towards the sum needed over £80,000 has been subscribed. One notes, too, the hard work of the Bradford Hospital Fund, which has resulted in 74 new firms having promised since January 1 to reinstate the system of collections.

Melbourne, Australia: The plans for the new blocks it is proposed to add to the hospital are now completed, and it is expected that tenders will be called for almost immediately, and the work put in hand. The new buildings are to be erected in sections so as to interfere as little as possible with the ordinary working of the hospital.

The new plans for the proposed isolation hospital at Hinckley, Leicestershire, which have been brought forward by Dr. Robinson, have been recommended for adoption by the Joint Hospital Committee. The chief feature of the new plans is that by greater means of ventilation more space will be saved. This space is to be used for the construction of a veranda and a third room, which together will accommodate nine patients. The veranda will be used for the open-air treatment of six cases.

### THE WEEK'S NOVELTIES.

In our advertisement columns will be found full particulars about the **HY-RIB STEEL LATH SYSTEM** which is worthy of careful consideration by architects and constructors. Hy-Rib is suitable for floors, ceilings, roofs, walls, partitions, furring, tanks, silos, and numerous other structures. It possesses a perfectly straight and true lath surface with expansion sufficient to provide a perfect clinch with the minimum amount of plaster. Special rolls of lathing are manufactured which can be bent to any desired arc of a circle varying from 13 in. to 20 ft. The Trussed Concrete Steel Co., Ltd., of Caxton House, Westminster (which has offices in all the principal cities) are the London agents for Hy-Rib, and will gladly send an illustrated pamphlet on the system to institutional workers who are interested in it.

We commend to the notice of institutional workers the **BERKEL'S PATENT SLICING MACHINE** particulars of which will be found in our advertising columns. This machine will especially appeal to those who are connected with hospitals, if for no other reason than because it ensures cleanliness and hygienic conditions in the slicing of food. After placing the substance to be cut on the machine it is only necessary to turn the fly wheel when beautiful slices of any desired thickness and uniform throughout are obtained. What, however, is equally important, is the fact that it abolishes waste in the kitchen, since it slices perfectly, whilst the time saved in cutting is enormous. The machine is fitted with a special hollow-ground, circular knife, on the razor principle, which ensures only the actual cutting-edge coming in contact with the article during slicing, and is also provided with an automatic sharpener, a most ingenious contrivance, by means of which a keen edge is always maintained. The importance of this will be fully appreciated when it is remembered how difficult it is to keep an edge on an ordinary hand knife, and the very poor and indifferent results too often obtained in consequence. The knife is, also, fully protected by a guard, which renders accidents to the operator impossible. The firm will be pleased to give a free practical demonstration to those interested, in order that they may have an opportunity of testing the machine, and seeing for themselves the great benefits and saving that accrue from its adoption. The machine is supplied in several sizes to meet all requirements, so that it is well within the reach of every institution, whether large or small.

We have received two of the beautiful gravure pictures which are now being given away on the coupon system, quite free of cost, to users of **OXO**, the well-known fluid beef. "The Return of the Hero" is one of the finest and most elaborate gift pictures ever presented to the public. It is reproduced on a tinted paper and mounted on a large sheet of heavy picture paper, plate sunk, and is equal in appearance and durability to pictures commonly sold in the art shops from two guineas upwards. It measures 40 by 33, and has a gravure surface of 27 by 18. The original was exhibited at the Royal Academy in 1909, and was painted by Fred Roe, R.I., who has exhibited historical works at the Royal Academy since 1887 and is well-known at the Paris Salon and all the principal exhibitions in this country. Mr. Roe painted the charming portrait of King Edward VII. for the King's Lynn Grammar School. The second picture is a little masterpiece by G. Sheridan Knowles, R.I., and was specially painted for the Oxo Company. Its title is "Blowing Bubbles," and it depicts a mother at play with two children amid charming rural surroundings. These pictures are among the most successful ever painted by artists, and are given free to users of Oxo for Oxo coupons to the face value of ten shillings and sixpence.

28 & 29 SOUTHAMPTON STREET, STRAND, LONDON, W.C.

### METROPOLITAN HOSPITAL-SUNDAY FUND

The Treasurers and Governors of all HOSPITALS, DISPENSARIES, and NURSING ASSOCIATIONS within the Metropolitan area, who desire that their Institutions shall participate in this year's DISTRIBUTION are hereby requested to send in their APPLICATIONS to the Secretary on or before Wednesday, the 1st March, 1911.

The Application must be accompanied by the Income and Expenditure Account and Balance Sheet for 1910, duly audited by a Public Accountant, together with Statistical Tables. The whole to be printed in accordance with the "Uniform System."

When these requirements have been complied with, the Committee of Distribution will send Forms, in duplicate, to the Secretaries of Institutions seeking to participate. One of these Forms must be fully and correctly filled in, and returned not later than Saturday, the 1st April, 1911.

The attention of the authorities of Institutions that did not participate last year is especially directed to the following law of the Fund :—

"That those Hospitals and Dispensaries only which are managed by a committee duly constituted, and which produce their printed Reports, with Income and Expenditure Accounts for the last THREE YEARS duly audited by a Public Accountant, in accordance with the uniform system of Accounts agreed upon, shall be allowed to participate in the Fund."

By order of the Committee of Distribution,

EDMUND HAY CURRIE, Secretary.

Mansion House, E.C., 1st February, 1911.

CHARTS THE BEST AND CHEAPEST PUBLISHED.

USED IN ALL THE PRINCIPAL HOSPITALS AND INFIRMARIES.

*SPECIMENS POST FREE.*

WODDERSPOON & CO., 6 GATE STREET, KINGSWAY, W.C.

The FUTURE of the HOSPITALS

*MEDICAL RELIEF FOR ALL ACCORDING TO MEANS.*

A Scheme of Co-operation between the PATIENTS, the HOSPITALS, and the STATE.

Price 2d. By Sir HENRY BURDETT, K.C.B., K.C.V.O. Price 2d.

THE SCIENTIFIC PRESS, Ltd., 28 & 29 Southampton Street, Strand, London, W.C.; and at all Bookstalls.

